# Computational modelling of γ-H2AX foci formation in human cells induced by alpha particle exposure

**DOI:** 10.1038/s41598-022-17830-8

**Published:** 2022-08-23

**Authors:** Ali Abu Shqair, Ui-Seob Lee, Eun-Hee Kim

**Affiliations:** Department of Nuclear Engineering, Seoul National University, Seoul, 08826 Republic of Korea

**Keywords:** Biophysics, Computational biology and bioinformatics, Engineering

## Abstract

In cellular experiments, radiation-induced DNA damage can be quantified by counting the number of γ-H2AX foci in cell nucleus by using an immunofluorescence microscope. Quantification of DNA damage carries uncertainty, not only due to lack of full understanding the biological processes but also limitations in measurement techniques. The causes of limited certainty include the possibility of expressing foci in varying sizes responding individual DSBs and the overlapping of foci on the two-dimensional (2D) immunofluorescence microscopy image of γ-H2AX foci, especially when produced due to high-LET radiation exposure. There have been discussions on those limitations, but no successful studies to overcome them. In this paper, a practical modelling has been developed to simulate the occurrences of double-strand breaks (DSBs) and the formations of γ-H2AX foci in response to individual DSB formations, in cell nucleus due to exposure to alpha particles. Cell irradiation and DSB production were simulated using a user-written code that utilizes Geant4-DNA physics models. A C +  + code was used to simulate the formation γ-H2AX foci, which were spatially correlated to the loci of DBSs, and to calculate the number of individual foci from the observed 2D image of the cell nucleus containing the overlapping γ-H2AX foci. The average size of focal images was larger from alpha particle exposure than that from X-ray exposure, whereas the number of separate focal images were comparable except at doses up to 0.5 Gy. About 40% of separate focal images consisted of overlapping γ-H2AX foci at 1 Gy of alpha particle exposure. The foci overlapping ratios were obtained by simulation for individual size groups of focal images at varying doses. The size distributions of foci at varying doses were determined with experimentally obtained separate focal images. The correction factor for foci number was calculated using the foci overlapping ratio and foci size distribution, which are specific to dose from alpha particle exposure. The number of individual foci formations induced by applying the correction factor to the experimentally observed number of focal images better reflected the quality of alpha particles in causing DNA damage. Consequently, the conventional γ-H2AX assay can be better implemented by employing this computational modelling of γ-H2AX foci formation.

## Introduction

Humans are exposed to diverse types of naturally existing or artificially generated radiations. Different types of radiation have different energy transfers per unit track length or different linear energy transfers (LETs). Charged particles of high LET resulting in dense energy deposition pattern along the track have a stronger impact of damaging bio-targets than photons of low LET. International Commission on Radiological Protection recommends the relative biological effectiveness (RBE) of alpha particles be twenty times that of X-rays^1^.

Genomic DNA in the cell nucleus is considered the most important target in living cells. Ionizing radiation damages the integrity of DNA by causing double-strand breaks (DSBs). DNA DSBs are critical to the cell fate. DSBs either would be correctly repaired or could trigger cell death or mutation^2,3^. A biomarker for experimentally detecting DSBs in a cell nucleus is the phosphorylated H2AX histones called γ-H2AX^4,5^. Gamma-H2AX molecules position and accumulate at DSB sites during a limited time period after DSB production, spread over large chromatin domains in the order of several megabase pairs (2 ~ 30 Mbp)^6^ near the DSB sites, and disappear following the DSB rejoining^7^.

Immunofluorescence microscopy is used to detect the focal images of immunostained γ-H2AX (γ-H2AX foci) and analyze the formation of γ-H2AX molecules in the cell nucleus. The amount and size of observed γ-H2AX foci change over time after irradiation^5,7,8^. Measurements of γ-H2AX foci are conducted mostly at a certain post-irradiation time corresponding to the peak count of γ-H2AX foci^6^. The number of γ-H2AX foci at the time of measurement does not necessarily equal the number of initially formed DSBs, as some γ-H2AX foci initially expressed in response to DSB formation might no longer be expressed at the time of measurement due to DSB repair^9^. Multiple DSBs can locate within the volume of a single γ-H2AX focus^10^. Foci of γ-H2AX are counted commonly from a two-dimensional (2D) immunofluorescence microscopy image of irradiated cells. Multiple overlapping foci projected on the 2D-image are indistinguishable from the conventional 2D-microscopy and can be detected as a single focus^11^.

Limited dimension and resolution of microscopical imaging would cause uncertainty in the measurement of DSBs, especially those in close proximity. Alpha particles position γ-H2AX foci close to their tracks, whereas X-rays form homogeneously sparse foci^12^. The expectation of a higher DSB yield by charged particles than by X-rays^13^ was challenged by the experimental observation of a similar number of or even less γ-H2AX foci from charged particle exposure than from X-ray exposure^14,15^. RBE of charged particles can be underestimated when only the number of γ-H2AX foci are considered. A recent study reported that relatively larger γ-H2AX foci were formed by alpha particle exposure than by X-ray exposure of the same dose^15^.

Several studies developed simulation scheme to reproduce experimental measurements of radiation-induced γ-H2AX foci. Barbieri et al.^11^ correlated the number of γ-H2AX foci counted from microscopical 2D-image with the DSB clusters simulated by PARTRAC code. They observed that DSB clusters increased with dose and thus γ-H2AX foci were more likely to merge in the 2D images, which can explain the saturation of the number of focal points at high doses. Vadhavkar et al.^16^ suggested that multiple DSBs in a repair domain would appear as a single γ-H2AX focus. Tommasino et al.^17^ modelled a spatially random spreading of γ-H2AX foci over up to 10 Mbp-sized domains around DSB sites.

In this study, a computational model was proposed to simulate the DSB production and γ-H2AX foci formation in human cells after alpha particle exposure. The simulation model was based on the experimental setup of previous work^15^. Measurement uncertainties due to the overlapping of projected γ-H2AX foci on the 2D-image in immunofluorescence microscopy were investigated using our model. A correction factor was calculated from the simulation results to obtain the actual number of γ-H2AX foci from observed 2D focal images in laboratory.

## Methods

### Modelling of cell exposure to alpha particles

**Figure 1:**
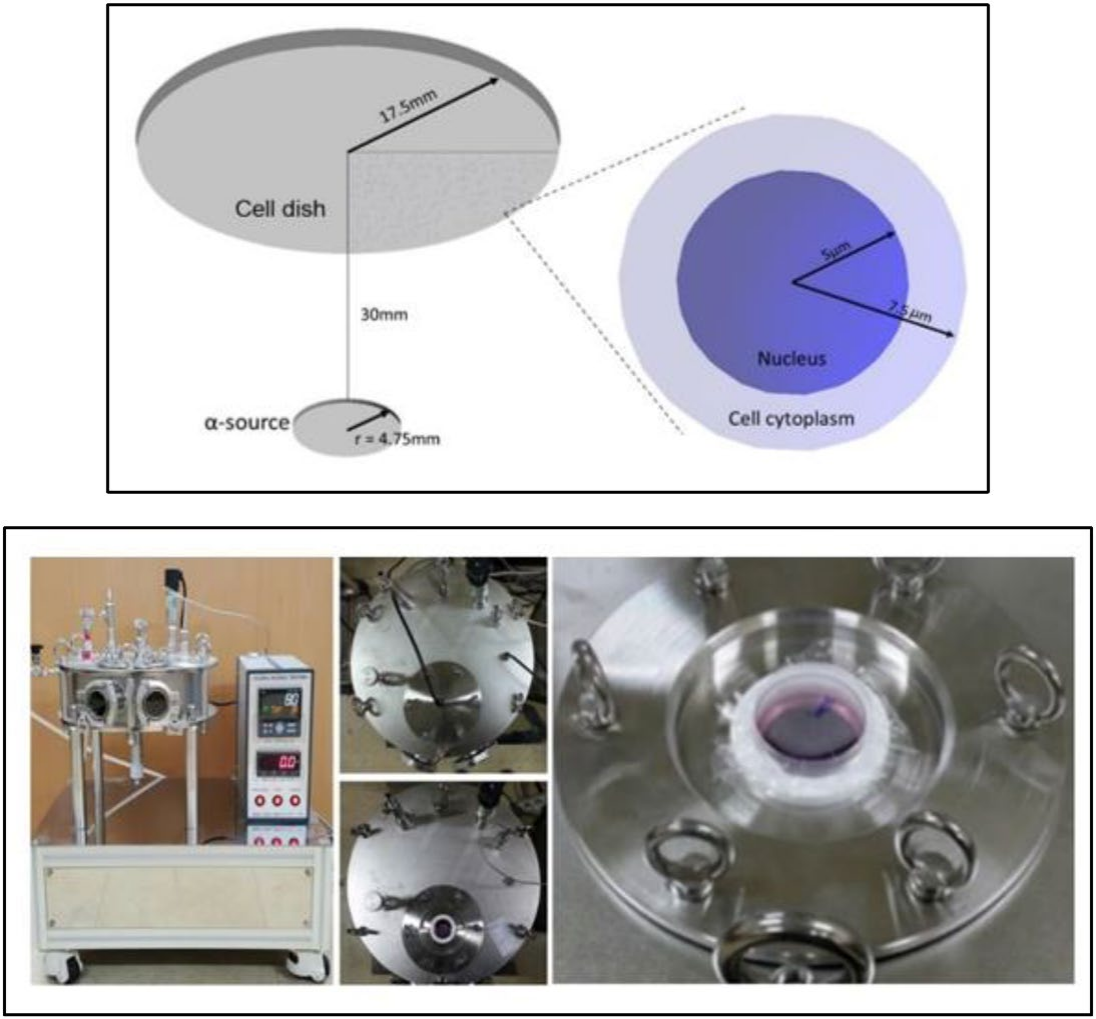
A geometrical model (left) for simulating cell exposure to alpha particles performed using the alpha particle irradiator (right) in the Radiation Bioengineering Laboratory at Seoul National University^18^: The bottom of Mylar cell dish, with cells on the top is placed in parallel over an alpha-emitting disc source.

Exposure of human lung epithelial cells (BEAS-2B) [Catalog No. CRL-9609, American Type Culture Collection (ATCC), Manassas, VA, USA] in a dish to alpha particles was modelled according to the setup of the alpha particle irradiator^18^ used in the Lee et al.’s experiments^15^. Figure 1 shows the geometry of the cell dish bottom and disc source modelled using the Geant4 toolkit^19^. Five thousand cells were randomly placed on the 4 μm-thick Mylar (Polyethylene Terephthalate) bottom of a 35 mm-diameter cell dish. The 9.5 mm-diameter Am-241 disc source emits alpha particles in random directions at a 30 mm of distance from the Mylar bottom. Alpha particles emitted towards the cell dish reach the dish bottom mainly with 5.48 and 5.44 MeV by the emission yields of 0.85 and 0.13 per decay, respectively^20^. Cells and culture medium were modeled as liquid water of 1.0 g/cm^3^ in density. The composition of Mylar was adopted from the database of National Institute of Standards and Technology.

### Simulation modelling of DSB production in cell nucleus

The size of simulated cell nuclei was based on the measurements of the 2D microscopic images of BEAS-2B cells. The nucleus area of 643 cells were calculated using the image analysis software “CellProfiler”^21^. Based on the average nucleus area (~ 79 ${\mathrm{\mu m}}^{2}$), cell nuclei were modeled as spheres of 10 $\mathrm{\mu m}$ in diameter. The nucleus was co-centered with a 15 $\mathrm{\mu m}$-diameter spherical cell. Geant4-DNA physics^22^ was used to simulate the track structure of alpha-particles in the nucleus and the Geant4 standard electromagnetic physics elsewhere outside the nucleus.

**Figure 2:**
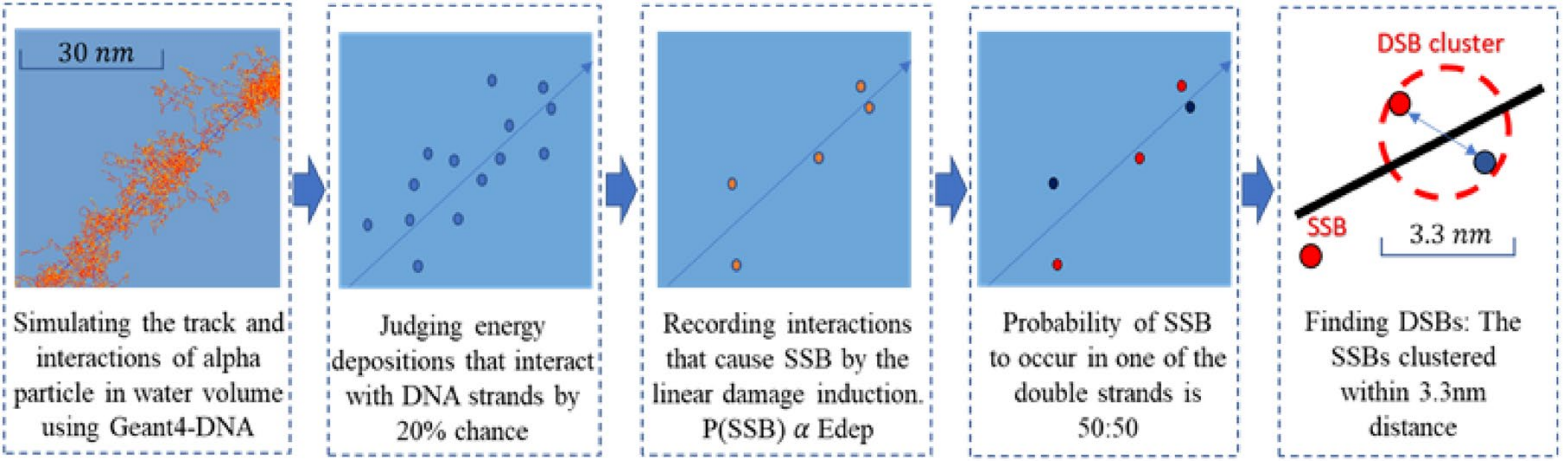
A computational scheme from alpha particle track simulation using GEAN4-DNA to DSB cluster registration using DBSCAN algorithm.

DSB production in cell nuclei was simulated using the damage-clustering algorithm developed in our previous study^23^. The algorithm was validated^23^ with its simulated yield of DSB production consistent with the simulation results by Friedland et al.^13^. The overall scheme from alpha particle tracking to DSB cluster registration is summarized in Fig. 2. Geant4-DNA physics enables simulating the energy deposition of alpha particles and secondary electrons on the nanometer scale^24,25^. The coordinates of energy depositions and absorbed doses in the hit nuclei were recorded for all alpha emissions. The coordinates belong to DNA matter by the probability equal to the volume fraction of DNA strands in the chromatin fibers. Damage to DNA strand from energy depositions, single-strand break (SSB), is determined by the probability of linear damage induction^26^, starting from zero for energy deposition below 5 eV and linearly increases until a value of 1 for 37.5 eV. A DSB cluster is formed by clustered SSBs, at least two in opposite strands, within a 3.3 nm in radial distance. DSB clusters were identified using the Density-Based Spatial Clustering of Applications with Noise (DBSCAN) algorithm^27,28^. Identified DSB clusters matter by the probability ${p}_{f}$ equal to the volume fraction of chromatin fibers in the nucleus. The spatial distribution of DSBs is highly correlated to the pattern of energy depositions. Unlike DSBs in space expanded from the main trajectory of X-rays, DSBs concentrate at small radial distances from the main trajectory of alpha particles. Simulation was performed for the number of alpha emissions (~ 10^8^) that result in 1 Gy of the average dose of cell culture medium.

### Modelling of statistical variation in γ-H2AX foci formation

The production yields of both DSBs and γ-H2AX foci linearly increase with the nucleus dose^29,30^. The number of γ-H2AX foci in the cell nucleus is proportional to the number of induced DSBs^29,31^. Practically, a focus formed due to DSB production may or may not still exist to be observed at the time of measurement after irradiation. The number of induced foci must be greater than that of measured foci.

The number of induced foci, an unknown parameter, could be obtained to fit the experimental measurements. We assumed that the γ-H2AX foci induced by DSBs are formed with a size that varies in a certain range and the size distribution is consistent regardless of the causative radiation type. The difference in size distribution due to the causative radiation type, as reported by Lee et al.^15^, was observed with the 2D images of foci from experiment. When cells are exposed to high-LET alpha particles, DSBs are clustered near the tracks and thus the induced γ-H2AX foci can overlap^12^. The sparse DSB productions by low-LET X-rays would result in separate γ-H2AX foci in cell nuclei. Different sizes of separate focal images obtained from X-ray exposure can be attributed to the statistical variation in foci formation.

**Figure 3:**
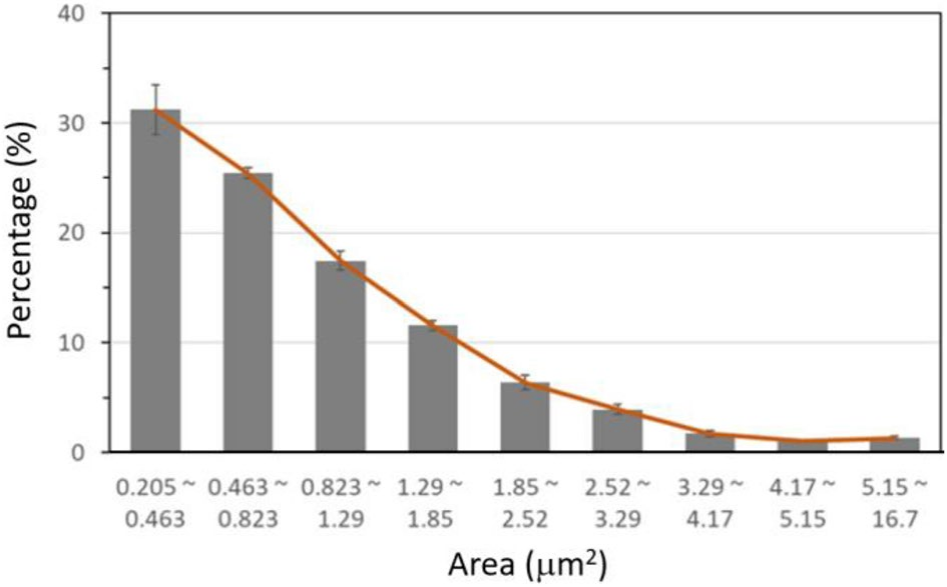
An approximate size-distribution function of γ-H2AX foci expressed in BEAS-2B cells 1 h after X-ray exposure. The distribution of discrete size groups (grey bars) were quoted from Lee et al.^15^.

Lee et al.^15^ measured the sizes of γ-H2AX foci expressed in BEAS-2B cells 1 h after X-ray exposure at doses varying from 0.1 to 1 Gy. The size distribution of γ-H2AX foci obtained by fitting the numbers of foci classified into different size groups was consistent regardless of dose^15^. Nevertheless, higher doses resulted in more foci formation and thus better statistics on the size distribution. Figure 3 shows the size distribution of γ-H2AX foci measured at 1 Gy of X-ray exposure, which suggests that DSB production can induce γ-H2AX focal image of a varying size.

### Simulation of γ-H2AX foci formation

In the experiments by Lee et al.^15^, γ-H2AX foci were viewed separate or overlapping in their 2D images. The separate foci formed along the trajectory of alpha particle in a cell nucleus can be observed to overlap if they are viewed in parallel direction to the track. Separate foci can be confirmed when they are viewed in the normal direction to the track. While going through the irradiation till observation in the experimental setup, cells were detached from the cell dish after immunostaining and attached to a slide glass via cyto-centrifugation. When viewed using a microscope, the alpha tracks in cell nuclei are oriented in random direction on the slide glass. Hence, the separate foci could be mostly viewed as separate because cells have little chance of being viewed in parallel direction to the alpha track. The overlapping foci in 2D microscopic image are probably the actual overlap.

**Figure 4:**
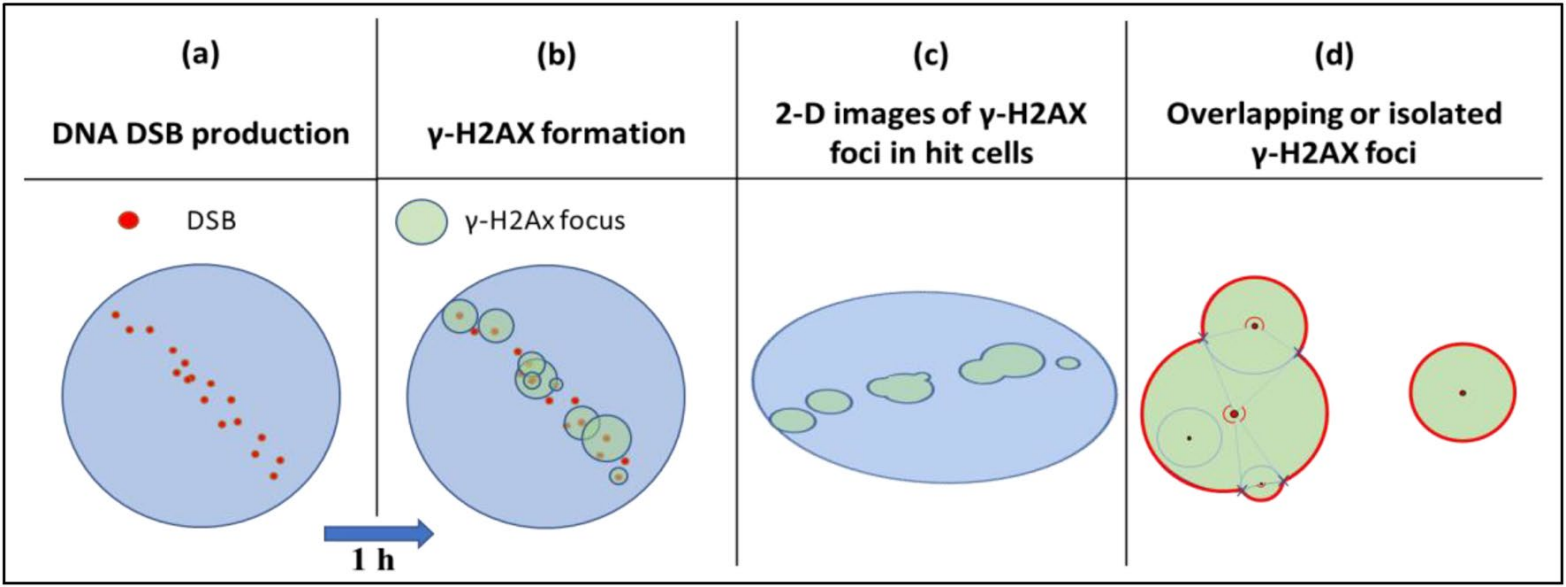
Illustration of γ-H2AX focal image analysis: (**a**) simulating DSB productions, (**b**) determining the sizes of γ-H2AX foci, (**c**) viewing the 2D images of γ-H2AX foci after cell rotation, and (**c**) identifying overlapping or isolated focal images.

Figure 4 depicts the procedure of simulating γ-H2AX foci formation in cells after alpha particle exposure by Monte Carlo method. The spatial distribution of energy deposition events were obtained by GEAN4-DNA simulation and DSB clusters were identified using the DBSCAN algorithm (Fig. 4a). For individual DSBs, γ-H2AX foci were formed with sizes randomly selected from the distribution in Figs. 3 and 4b. Individual foci may be isolated from others or overlap depending on their sizes and distances. The 2D microscopic view of γ-H2AX foci in cells placed on the slide glass in random direction after alpha particle exposure was simulated by rotating all the irradiated cells around their centers with arbitrary azimuthal and polar angles (Fig. 4c). The γ-H2AX focal spots after cell rotation have different 2D coordinates from those at formation. Individual focal images may consist of a single γ-H2AX focus or multiple overlapping foci that intersect each other in a 2D projection (Fig. 4d).

A computational algorithm was developed to analyze the multiplicity of γ-H2AX foci within individual focal images from the 2D projections, obtained in the experiments of Lee et al.^15^ and in this simulation. The γ-H2AX focal image of a simple circular form was identified as a single isolated focus whereas the image of a domain enclosed by more than two arcs of different radii of curvature was identified to contain overlapping foci. The total number of individual foci induced in a cell nucleus was calculated by decoupling the overlapping foci in each focal image.

## Results and discussion

### Monte carlo estimates of DSB production

**Figure 5:**
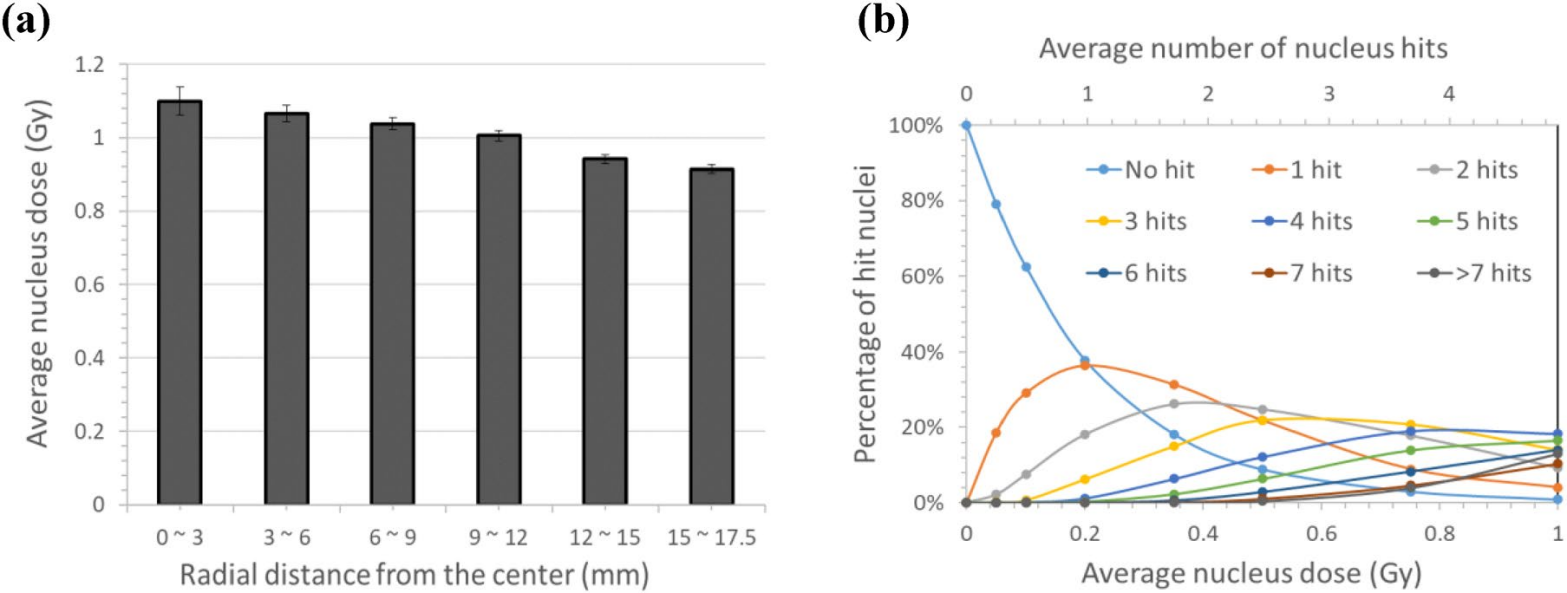
Results from GEANT4-DNA simulation: (**a**) average nucleus doses at different radial distances from the dish center, and (**b**) probabilities for the number of nucleus hits varying with the average nucleus dose.

The average LET of alpha particles hitting cells in vitro was about 100 keV/μm. A slight variation in dose was observed among the cells. Figure 5a shows the average nucleus dose varying with the radial distance from the dish center at the average 1 Gy of culture medium exposure to alpha particles. Nucleus dose differed by 17.5% between the cells near the center (0 ~ 3 mm) and the cells near the edge (15 ~ 17.5 mm). For the spherical cells with nominal diameter of 15 μm spread on the Mylar bottom, the average number of nucleus hits was 4.9. Figure 5b shows the probabilities for possible number of nucleus hits at different average nucleus doses up to 1 Gy. The average number of nucleus hits calculated from the probabilities specific to the average nucleus dose is marked on the upper axis, which increases with the average dose. At 0.1 Gy of medium dose, about 60% of cell nuclei are not hit and 30% of cell nuclei are hit once. As the average dose increases, a smaller portion of cell nuclei are hit-free.

### Monte carlo estimates of γ-H2AX foci formation

**Figure 6:**
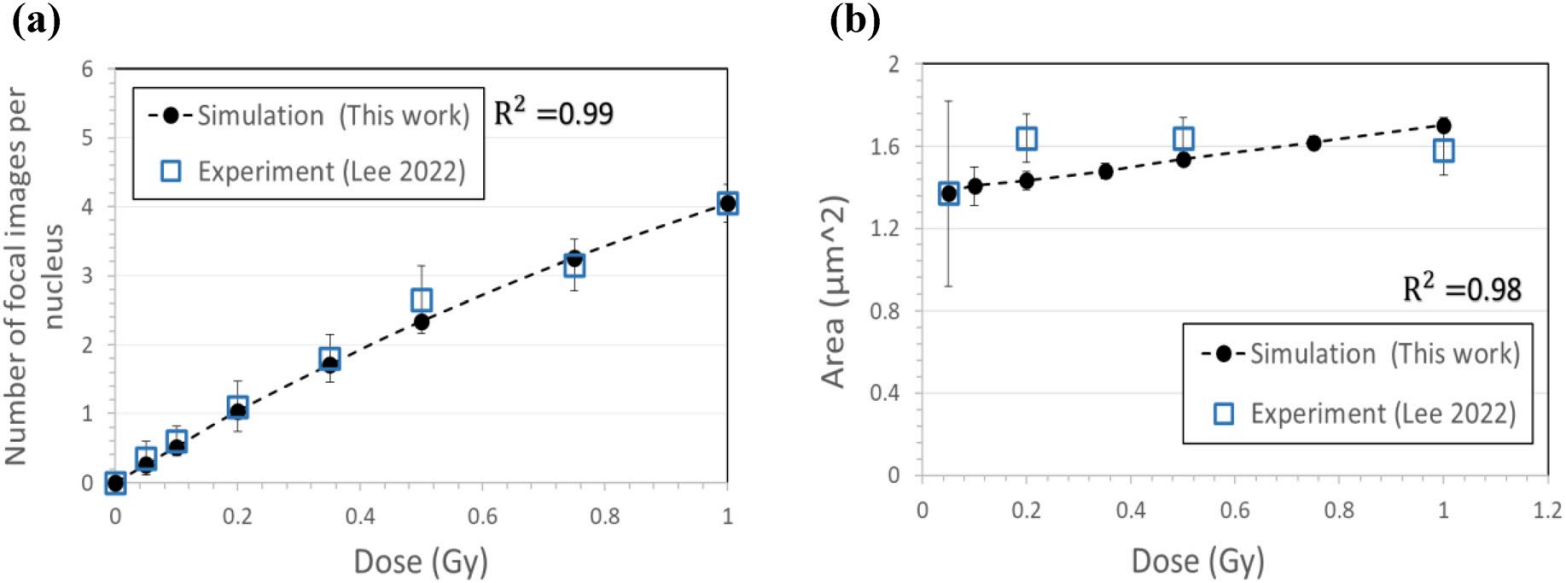
Estimates from Monte Carlo simulation compared with those from experimental measurement by Lee et al.^15^: (**a**) average number and (**b**) average area of γ-H2AX focal images in BEAS-2B cells 1 h after alpha particle exposure.

Simulations of γ-H2AX foci formation were performed, with different assumption of the yield of γ-H2AX foci formation per dose in each simulation. Twenty independent simulations were performed for each assumption. The average number and area of focal images in 2D projection were compared to the measurements obtained from four independent experiments. The best fit of simulational results to experimental measurements at all doses up to 1 Gy was obtained at a yield of 8 foci/Gy. Figure 6 depicts the results of our Monte Carlo simulation compared to the experimental measurements by Lee et al.^15^.

Simulational results and experimental measurements show good fit with R^2^ greater than 0.99 for average number of γ-H2AX focal images (Fig. 6a). The number of focal images increases with the dose, but by less degree per dose increment as dose increases. Given that the average yield of γ-H2AX foci formation per dose remains constant, Fig. 6a implies that a greater portion of focal images consist of overlapping foci as dose increases. Experimental measurements do not imply significant change in the mean size of γ-H2AX focal images with the nucleus dose whereas the mean size of focal images is expected to increase with the dose according to the simulation results (Fig. 6b). Given that the average yield of γ-H2AX foci formation per dose remains constant, Fig. 6b implies that overlapping foci are more concentrated in a cell nucleus as dose increases.

**Figure 7:**
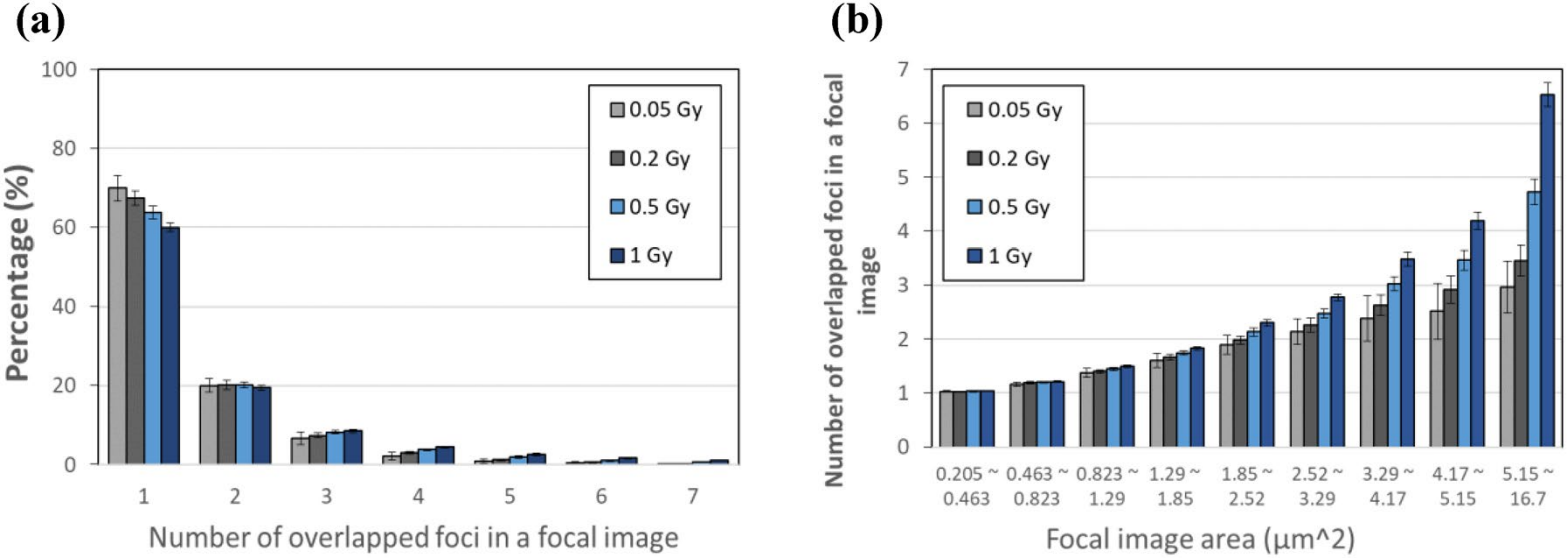
The multiplicity distribution of γ-H2AX foci in a single focal image induced at 0.05, 0.2, 0.5 and 1 Gy of alpha particle exposure: (**a**) the percentage of focal images that consist of multiple overlapped foci, (**b**) the average number of overlapping foci in a focal image of different sizes.

Figure 7 depicts the distributions of γ-H2AX foci multiplicity per focal image, obtained from simulation at different doses. The portion of focal images consisting of multiple overlapping foci increases with the nucleus dose, about 30% at 0.05 Gy increasing to 40% at 1 Gy (Fig. 7a), which is consistent with the implication of Fig. 6a. A larger focal image was attributed to more foci overlapping, even though there was a chance of a larger isolated focal image than a focal image of overlapping foci according to the stochastic variation in size of induced γ-H2AX focus (Fig. 3). A focal image of given size has more overlapping foci at a high dose than at a low dose (Fig. 7b), which is consistent with the implication of Fig. 6b. For instance, an observed γ-H2AX focal image of 0.463 ~ 0.823 ${\mathrm{\mu m}}^{2}$ in cells irradiated at 0.05 and 1 Gy consisted of 1.16 and 1.2 overlapping foci on average, respectively. A focal image of 5.15 ~ 16.7 ${\mathrm{\mu m}}^{2}$ consisted of 3 and 6.5 overlapping foci on average from exposures at 0.05 and 1 Gy, respectively.

### Application of simulation estimates

Calculated distribution of foci multiplicity (Fig. 7b) can be used to correct the number of observed focal images in laboratory for the actual number of induced γ-H2AX foci. In addition to the average number and area of γ-H2AX focal images in BEAS-2B cells (Fig. 6), Lee et al.^15^ calculated the ratio of focal images in nine discrete size groups ranging from 0.205 to 16.7 ${\mathrm{\mu m}}^{2}$, which agree with the size groups in Fig. 7b, at doses of 0.05, 0.2, 0.5, and 1 Gy from alpha particle exposure. Simulation results (Fig. 7b) were utilized to derive dose-specific correction factors and to calculate the actual number of induced γ-H2AX foci by applying the correction factors to experimental data of focal images observed by Lee et al.^15^ as follow:

1 $${\text{ correction}}\;{\text{factor}}\;\left( {\text{D}} \right) = \mathop \sum \limits_{i = 1}^{{9}} \left( { {\text{multiplication}}\;{\text{factor}}\;\left( {{\text{D; }}A_{i} } \right)_{{{\text{sim}}}} \times {\text{ratio}}\;{\text{of}}\;{\text{foci}}\;{\text{(D; }}A_{i} {)}_{{{\text{exp}}}} } \right)$$

2 $$\begin{aligned} {\text{average}}\;{\text{number}}\;{\text{of}}\;{\text{foci}}\;\left( {\text{D}} \right) & = {\text{correction}}\;{\text{factor}}\;\left( {\text{D}} \right) \\ & \quad \times {\text{mean}}\;{\text{number}}\;{\text{of}}\;{\text{focal}}\;{\text{images}}\;\left( {\text{D}} \right)_{{{\text{exp}}}} \\ \end{aligned}$$

where *i* is the index for the size group of γ-H2AX focal images.

**Figure 8:**
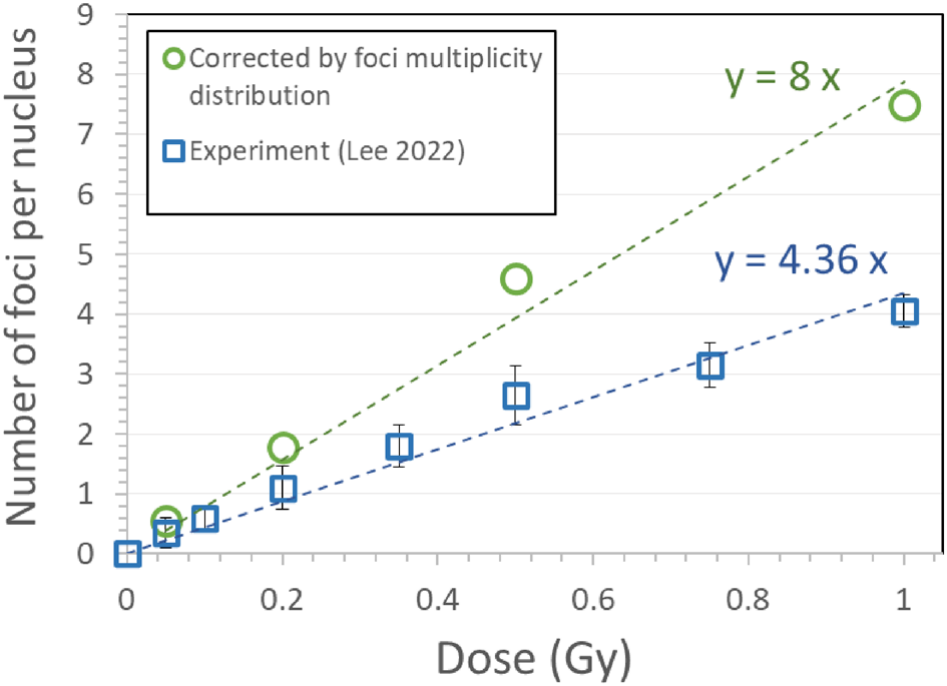
Mean number of γ-H2AX foci per nucleus in BEAS-2B cells exposed to alpha particles: as observed in experiments by Lee et al.^15^ (squares) and corrected by utilizing the multiplicity distribution of γ-H2AX foci in a single focal image (circles).

Figure 8 presents the mean number of γ-H2AX focal images per nucleus observed by Lee et al.^15^ from alpha particle exposure at different doses in comparison with the numbers of γ-H2AX foci estimated by correcting for the foci overlapping in nucleus. The average correction factor over different doses was about 1.8, which explains that about 46% of γ-H2AX foci were not counted due to overlap of 2D focal images.

### Application and limitations

In this study, we evaluated the underestimation of DNA damage due to the overlapping of γ-H2AX foci on 2D immunofluorescence microscopy image after alpha particle exposure. The estimated RBE depending only on the number of separate focal images would be about 0.9 while the corrected RBE is 1.8. Results are in general agreement with prior studies^11,16^. Barbieri et al.^11^ calculated the expected number of separate focal images from clustered DSBs within a specific radial distance (0.5 and 1 μm). Vadhavkar et al.^16^ considered the number of repair domains, consisting of a voxel with one side of 1.55 μm where enclosed DSBs are merged to form a single γ-H2AX focus.

Our modelling differs in that it adopts a distribution of statistically varying γ-H2AX focal size and considers both the number and area of separate focal images for the comparison with experimental observation. The motivation for adopting varying sizes of foci expression was that the size distribution of foci after X-ray exposure reported by Lee et al.^15^ can not be computationally reproduced by the clustering of equal-sized foci. DSBs formed by exposure to X-rays are homogeneously sparse^12^ and clustered DSBs leading to foci overlap is unlikely. We infer that the overlapping increases as the particle LET increases because the number of induced γ-H2AX foci is expected to increase due to many DSBs occurring close to the main track.

The 1 h post-irradiation of observation time in this study was the least elapsed time after irradiation until the irradiated cells are ready on a slide for microscopy. The size distribution of γ-H2AX foci 1 h after X-ray exposure was utilized in simulation to randomly select the size of γ-H2AX foci formed in response to DSB production. The repair of DSBs and thus the reduced number of γ-H2AX foci for 1 h since foci formation were not considered when the simulation data were fitted to the experimental measurements. The overlapping foci represent the complex DSBs, whose repair proceeds slowly^32^. Hence the foci overlapping measured 1 h post-irradiation is expected to be about the same as initially formed.

The variation in cell size and radio-sensitivity for DSB production during the cell cycle was not considered in this study. The cell size does not make any difference in simulation, but the nucleus size affects the energy deposition and the DSB production. In this study, the size of nucleus was approximated to the average size of cell nuclei that was measured in our previous work^15^ performed without cell synchronization. The correction of the number of foci was made by comparing the simulation data and the experimental observation for the same cell types. The correction factor may differ depending on the cell cycle distribution of test cells, but the algorithm correcting measurement for foci overlapping remains valid.

This study is essentially useful for RBE estimation as the multiplicity distribution of γ-H2AX foci formation is a measure of the complexity of DSB production. The complex DSBs are slowly repaired^32^ and more error-prone than simple DSBs^33^, which enhances RBE.

## Conclusion

This study modelled the production and measurement of DSBs and γ-H2AX foci in human cells after exposure to alpha particles. Uncertainties caused by the overlapping of induced γ-H2AX foci and their 2D projections in microscopy were investigated. The overlapping was serious when cells were irradiated with high-LET charged particles, because DSBs were concentrated along the particle tracks. The ratio of focal images that consisted of overlapping foci reached 40% at 1 Gy of alpha particle exposure. The corrected yields of γ-H2AX foci formation per cell from alpha particle exposure better fit to the expectation of high relative biological effectiveness from alpha particle exposure.

## Data Availability

The datasets used and/or analysed during the current study available from the corresponding author on reasonable request.
